# A rapid and low-cost handheld method for measuring the mean leaf inclination angle of crop canopies

**DOI:** 10.1016/j.plaphe.2026.100260

**Published:** 2026-07-22

**Authors:** Masaki Okamura, Ryo Yamazaki, Natsumi Okamura, Yumi Shimazaki, Ritaro Sato, Ryutaro Morita, Naohiro Aoki

**Affiliations:** a Central Region Agricultural Research Center, NARO, 1-2-1, Inada, Joetsu, Niigata, 943-0193, Japan; b Institute of Crop Science, NARO, 2-1-2 Kannondai, Tsukuba, Ibaraki, 305-8518, Japan; c Graduate School of Agricultural and Life Sciences, The University of Tokyo, 1-1-1, Yayoi, Bunkyo-ku, Tokyo, 113-8657, Japan

**Keywords:** Leaf orientation, Leaf angle, Canopy photosynthesis

## Abstract

Leaf inclination angle (LIA) strongly affects canopy photosynthesis and is an important trait for crop productivity. Difficulty in determining mean LIA throughout the canopy makes it a trait rarely evaluated in crop science research. Here, we report a novel, low-cost, simple method to rapidly measure the mean LIA throughout the canopy. We captured RGB and depth images of the crop canopy with a compact stereo depth camera aligned parallel to the ground in a handheld manner. The mean LIA was estimated through simple vector calculations. To validate the accuracy of this method, we used tests with a white board fixed at known angles and single leaves whose angle had been measured with a digital level. The mean LIA was strongly correlated with the mean tilt angle—a trait similar to LIA that was measured with a plant canopy analyzer, an expensive instrument for measuring leaf area index. Using our method, we were able to detect differences in LIA among cultivars, growth stages, and plant densities in rice, barley, soybean, and wheat. Because this method is very easy and has minimal financial, time and labor requirements, it is likely to be highly beneficial for crop scientists whose primary research is conducted in fields.

## 1 Introduction

Leaf inclination angle (LIA) strongly affects canopy photosynthesis and is an important trait for crop productivity. In the model of canopy photosynthesis by Monsi and Saeki [1], plant dry matter production is determined by the light-assimilation curve of a single leaf, the leaf area index, and the light extinction coefficient (*k*). Mean LIA throughout a canopy is the factor most influencing *k* in their model, which assumes that all leaf elements in the canopy are identical and are distributed randomly. However, difficulty in measuring mean LIA throughout the canopy makes it a trait rarely evaluated in crop science research. The mainstream approach for measuring LIA still relies on accelerometers or a protractor to determine the angles of individual leaves in field experiments [2,3]. However, this approach does not reflect the mean LIA of the canopy and may be subject to arbitrary bias in the selection of leaves for measurement.

Methods for measuring LIA in plants by using three-dimensional (3D) sensing have been developed rapidly in recent years, but many of them are for indoor use [4,5] or require a large-scale system [6]. Methods using LiDAR have been reported for outdoor trees [[7], [8], [9], [10]] and wheat [11]. However, it is difficult to say that 3D-sensing techniques have reached researchers in crop science, who are not specialists in remote sensing. One likely reason is that applying these methods to crop canopies requires the measurement device to be fixed in the field in some manner. In crop science, where several repeated measurements are necessary to account for spatial heterogeneity within a field, the installation and relocation of such equipment would demand substantial time and labor. From the perspective of crop scientists, LIA is only one of numerous field-measured traits, and therefore it is very important that they measure it inexpensively and with minimal labor.

Uto et al. [12] reported that the relative distribution of leaf angles within a crop canopy can be captured with a compact and inexpensive stereo depth camera. They regarded the depth differences between pixels, excluding background and discontinuous regions, as values related to leaf angle and reported that relative differences in canopy LIA could be captured. However, this study has several limitations: (1) validation of accuracy was limited to a qualitative comparison based on visually observable differences in leaf angle associated with the presence or absence of silicate fertilizer; (2) absolute leaf angle values were not quantified; (3) changes in pixel-level projected area resulting from differences in leaf angle were not accounted for; and (4) the camera is mounted on some form of support during measurement, and it remains unclear whether LIA can be measured easily in a handheld manner. For these reasons, the method cannot be considered a fully developed technique for measuring mean LIA of a canopy.

Here, we further developed their method to measure the absolute mean LIA throughout the canopy by using an inexpensive, compact stereo camera and a laptop computer in a handheld manner. Using absolute 3D coordinates that account for the camera's angle of view, this method successfully computes a weighted mean LIA that incorporates pixel-level area differences through simple vector calculations. It provides sufficient accuracy to capture the differences among cultivars, growth stages, and plant densities in mean LIA in field crop canopies.

## 2 Materials and methods

### 2.1 Capturing images and calculating mean LIA

**Fig. 1:**
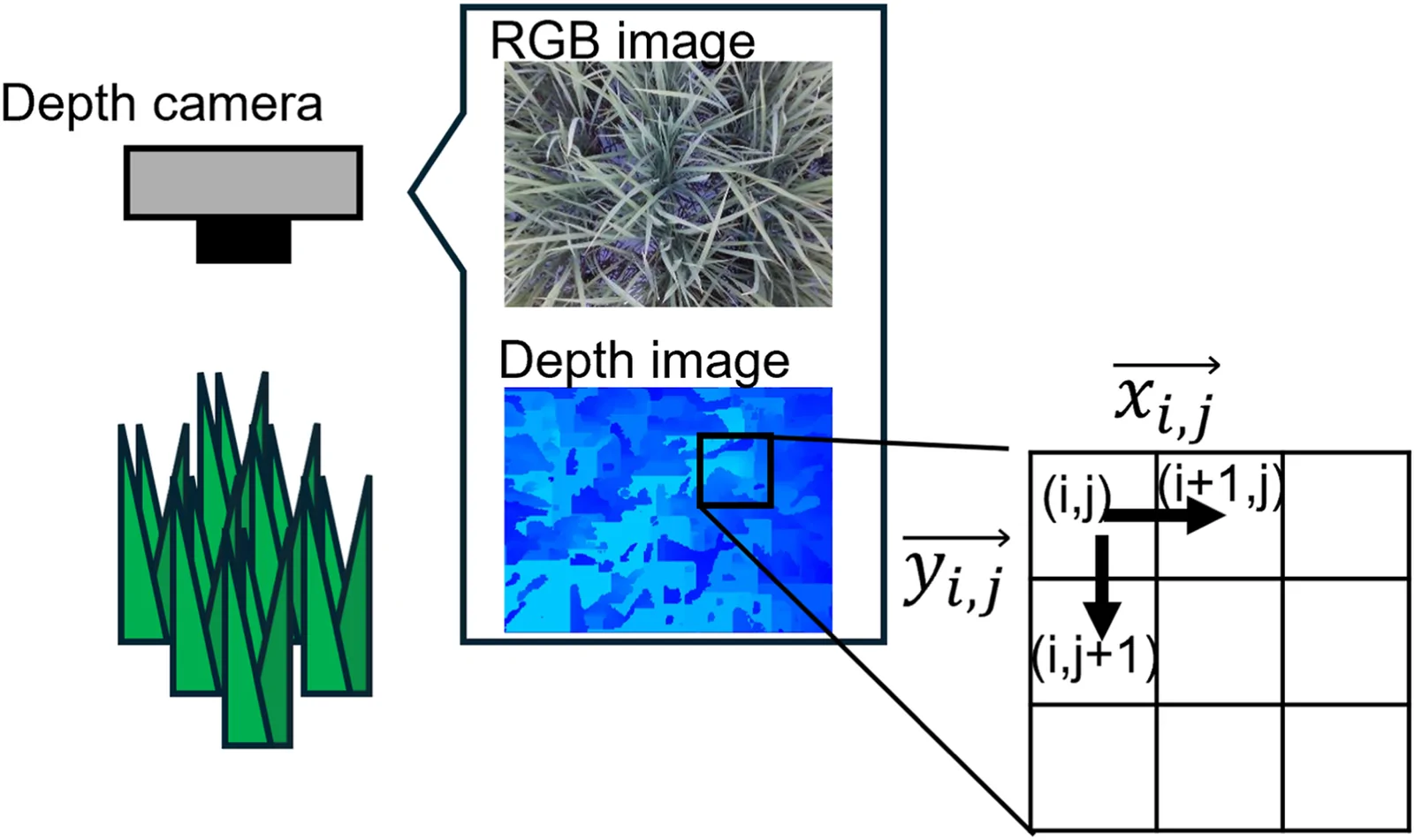
Method for measuring mean leaf inclination angle (LIA). RGB and depth images of the crop canopy were captured with a stereo depth camera aligned parallel to the ground in a handheld manner. The mean LIA was estimated through simple vector calculations.

RGB and depth images of the crop canopy were captured at a resolution of by 640 × 480 pixels and 30 frames per second (color format: BGR8; depth format: Z16) using a stereo depth camera (D435if, Intel, Santa Clara, CA, USA) aligned parallel to the ground in a handheld manner (Fig. 1). Depth data were post-processed using the RealSense SDK 2.0 library (Intel) in Python v. 3.9. Specifically, a spatial filter was applied with filter magnitude set to 1, smooth alpha set to 0.25, and smooth delta set to 50. In addition, a hole-filling filter was applied using the default parameters. Other processing options were not configured and therefore remained at the SDK default settings.

**Fig. 2:**
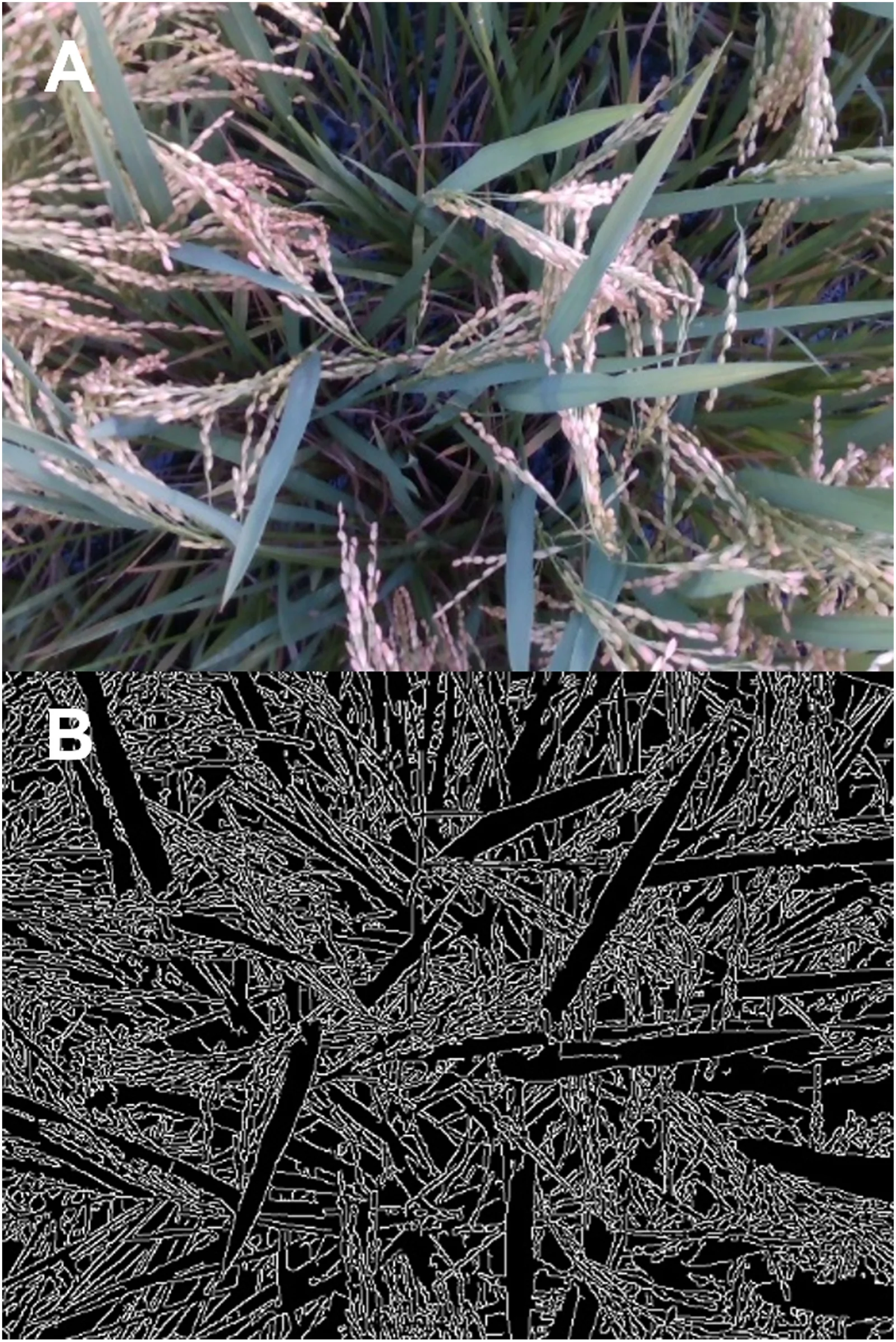
An RGB image captured by the depth camera (A) and identified edges (B).

The relative 3D coordinates of each pixel (*i*, *j*) in the captured images were obtained by using the pyrealsense2.rs2_deproject_pixel_to_point() function from the RealSense SDK 2.0 library. Spatial vectors (**x**_***i*,*j***_ and **y**_***i*,*j***_) were calculated from the coordinate differences between adjacent pixels in the *x*- and *y*-directions. The area of the parallelogram formed by these two vectors (computed via the cross product) was defined as the pixel area *S*_*i,j*_, and the angle between this parallelogram and the vertical direction was defined as the LIA *θ*_*i,j*_. A base height was defined according to the size of the plants and measurement targets, and pixels located below this threshold (close to the ground) were excluded from the subsequent calculations. RGB images were first converted to grayscale images using the cv2.cvtColor() function from the OpenCV library (OpenCV team), and edges were subsequently detected using the cv2.Canny() function. The lower and upper thresholds for edge detection were set to 20 and 120, respectively. Pixels identified as edges and those within ±3 pixels in both the *x*- and *y*-directions were excluded from the subsequent calculations. This procedure allowed us to exclude pixels corresponding to leaf margins and panicles after heading in rice (Fig. 2). The upper and lower 10% of *θ*_*i,j*_ were removed as outliers, and the mean LIA was calculated by using the following weighted-average formula:

$$Mean\;LIA=\sum_{\left(i,j\right)\in \Omega }\left(S_{i,j}\times \theta_{i,j}\right)/\sum_{\left(i,j\right)\in \Omega }S_{i,j}$$

where *Ω* denote the set of pixels that remain after the removal processing by base height and edge detection. This method is patented in Japan (JP Patent 7749275), and the associated Python program is distributed under a licensing agreement with the National Agriculture and Food Research Organization (NARO).

### 2.2 Accuracy verification test

**Fig. 3:**
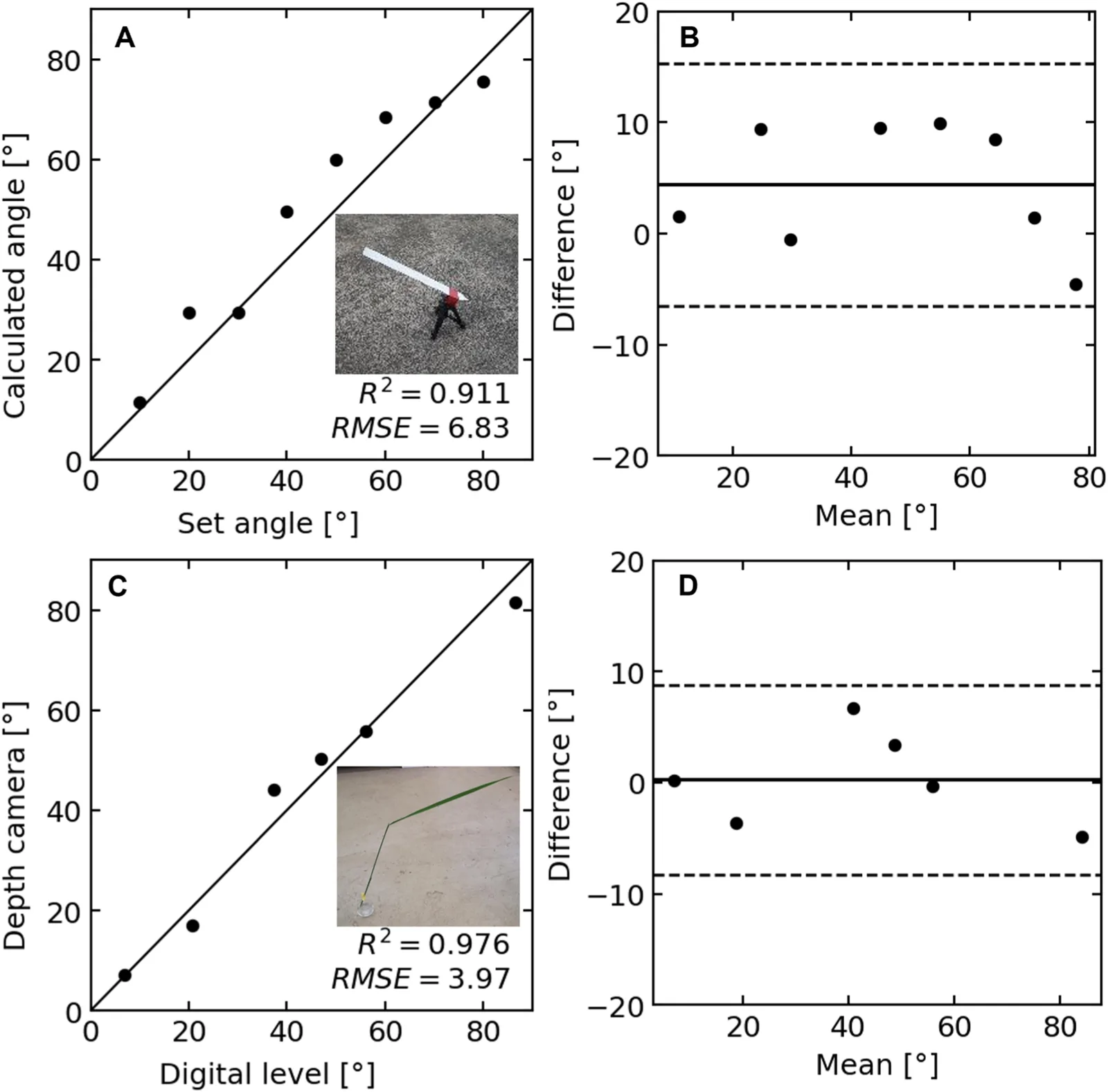
Validation test for measuring the accuracy of leaf inclination angles. (A) Comparison between the set angle of a white board and angle calculated from images taken with a depth camera. (B) Bland-Altman plot for the white board test. (C) Comparison between single-leaf angle measured by a digital level and those calculated from images taken by a depth camera. (D) Bland-Altman plot for the single-leaf test. Diagonal lines indicate 1:1. *R*^*2*^, coefficient of determination. *RMSE*, Root Mean Square Error. The solid horizontal line represents the bias (mean difference), and the dashed lines represent the limits of agreement (bias ± 1.96 × the standard error of the bias).

A white board was fixed at angles from 10° to 80° in 10° increments, and the mean LIA was measured (Fig. 3A). Next, the mean LIA of a single unbent leaf was measured (Fig. 3C). The angle near the center of the leaf was measured with a digital level (DGL-C, Myzox, Aichi, Japan) and then with the depth camera. The base height was set at 0.3 m, and the measurement height was 1.0 m.

### 2.3 Field experiments with rice

Rice (*Oryza sativa* L.) cultivars ‘Koshihikari’ (*japonica*), ‘IR64’ (*indica*), and near-isogenic lines (NILs) KH-TAC1, KH-LIA3, and IR64-tac1 were used. KH-TAC1 [13] has spread tiller angles, whereas KH-LIA3 [2] has erect LIA; both are in the genetic background of ‘Koshihikari’. IR64-tac1 [13] is a reciprocal NIL with erect tiller angles in the genetic background of ‘IR64’.

Seedlings (21 days old) were transplanted on 14 May 2024 at a density of 22.2 hills m^−2^ (spacing of 15 cm × 30 cm) with one plant per hill. They were grown in an experimental paddy field at the Hokuriku Research Station of NARO (37°12′N, 138°27′E; 9.5 m above sea level), Joetsu, Niigata, Japan. Plots (4.05 m^2^ each) were arranged in a randomized block design with three replicates. Controlled-release fertilizer (LP40: LPs100 = 1:2; JCAM Agri, Tokyo, Japan) was applied as a basal N dressing at 5 g N m^−2^. LP40 and LPs100 release 80% of their total N content at 25 °C within 40 and 100 days, respectively, after application. Inorganic fertilizers were applied as a basal dressing at 6 g P m^−2^ and 5 g K m^−2^.

All parameters were measured five times during the growing period on the same dates for all plants, except at the full heading stage (9 August for Koshihikari, KH-TAC1, and KH-LIA3 and 16 August for IR64 and IR64-tac1). The tiller angle was measured between the tiller and a vertical line with the DGL-C digital level at each time point. Three reproductive tillers per hill were measured, and the mean of two consecutive plants per plot was calculated as the tiller angle of the plot.

Mean tilt angle (MTA) was measured with a plant canopy analyzer (PCA; LAI-2200C, LI-COR, Lincoln, NE, USA). In each replication, we took three series of measurements in rapid succession. One series consisted of one measurement above the canopy and four measurements below the canopy (at the water surface). Measurements were taken during cloudy weather with no direct sunlight.

Mean LIA was also measured with the D435if depth camera immediately after MTA measurement, with one point per plot. The base and measured heights were 0.1 m and 0.8 m, respectively, on 25 June; 0.1 m and 1 m on 16 July; and 0.2 m and 1.2 m after full heading.

### 2.4 Field experiments with barley

Barley (*Hordeum vulgare* L.) cultivar ‘Fiber-Snow’ was sown in a drained paddy field at the Hokuriku Research Station of NARO on 3 October 2025. Inorganic fertilizers were applied as a basal dressing of 6 g N m^−2^, 6 g P m^−2^, and 6 g K m^−2^. The high (9.2 g m^−2^) and low (4.8 g m^−2^) seeding density plots were arranged in a randomized block design with three replicates, each with a plot size of 9.6 m^2^. Ammonium sulfate was applied as top dressing before winter at 4 g N m^−2^ on 4 November. On 11 December, mean LIA was measured, with three measurement points per plot. The measurement height was 0.8 m and the base height was not set because the plants had rosette leaves, and leaves touching the ground needed to be included at this growth stage. Although this method includes the ground surface in the angle calculation, we judged that its influence was minimal because the canopy coverage ratio was sufficiently high.

### 2.5 Statistical analysis

Tukey's multiple comparison test was conducted with the glht() function from the multcomp library [14], and two-way analysis of variance (ANOVA) was conducted with the aov() function in R v. 4.3.1 software (R Foundation). Student's *t*-tests were conducted with the ttest_ind () function from the SciPy library (SciPy Project Team) in Python v. 3.9.

## 3 Results

### 3.1 Validation using objects with known angles

We fixed a white board to imitate a leaf at arbitrary angles and used a depth camera to measure the mean inclination angle of this board. Our preliminary tests indicated that the depth images acquired by the stereo depth camera used in this study contained a number of outliers. Therefore, to improve the accuracy of mean LIA estimation, a procedure for outlier removal was applied. A sensitivity analysis to determine the appropriate outlier range was conducted. The analysis showed that excluding the upper and lower 5% or 10% of values improved measurement accuracy (Supplementary Fig. S1). Based on this result, in subsequent analyses, the upper and lower 10% of values were treated as outliers and excluded when calculating mean LIA. The calculated angles of this method closely matched the set angles, with only a small bias (Fig. 3A and B). Next, we isolated a single rice leaf, positioned it so that the mean LIA could be measured by using a digital level, and compared the data with those obtained by using a depth camera. The two methods showed good agreement, with almost no bias (Fig. 3C and D).

### 3.2 Practical applications in rice

**Fig. 4:**
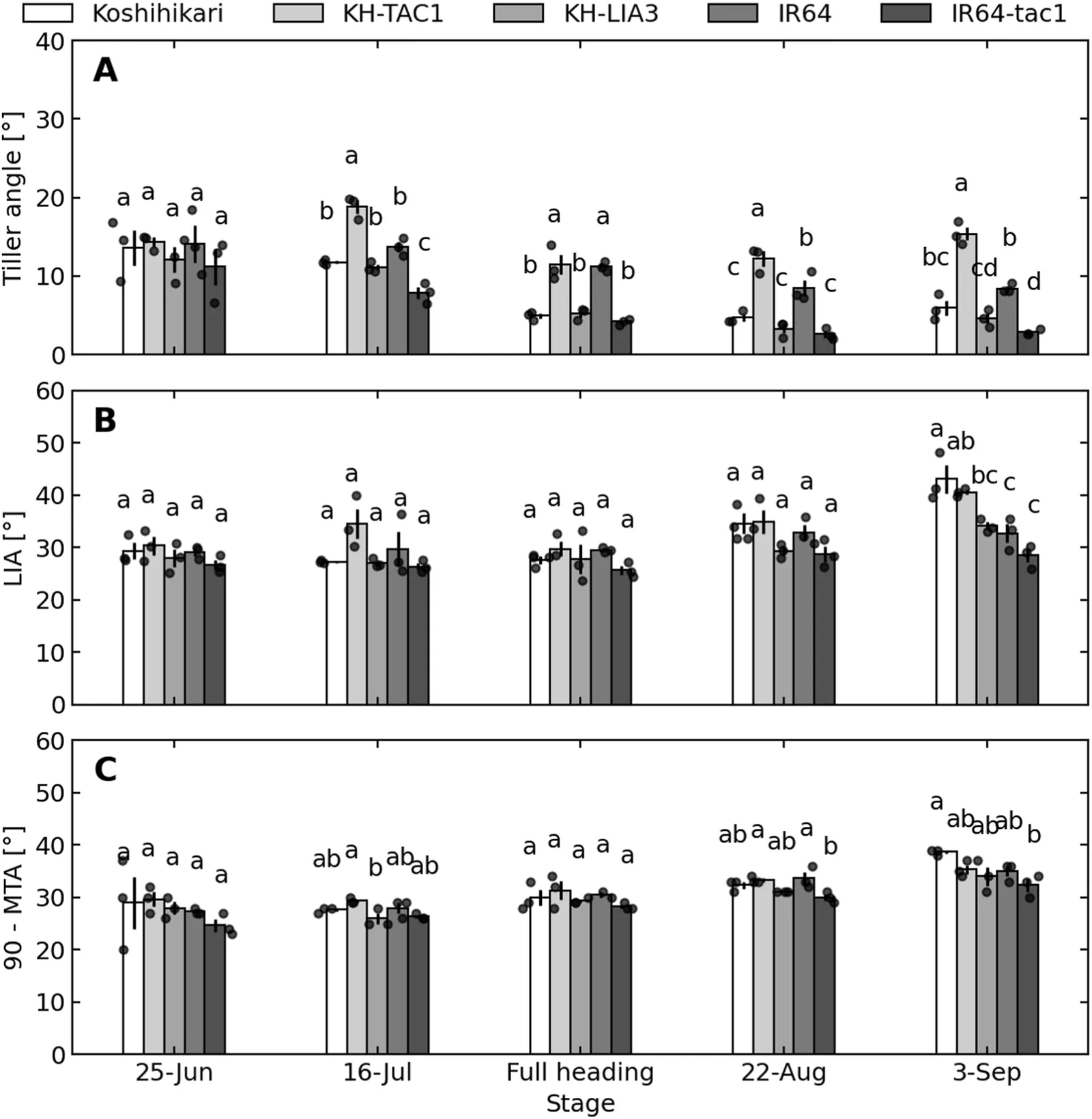
Tiller angle (A), leaf inclination angle (LIA) measured by a depth camera (B), and mean tilt angle (MTA) measured by a plant canopy analyzer (C) in rice. Values are means ± standard error (n = 3), with individual data points overlaid. Bars with a common letter within a cultivar/line are not significantly different (*P* ≥ 0.05, Tukey's test).

In a crop canopy, leaves are oriented in various directions and are often bent, making the measurement of mean LIA challenging. To address this challenge, we validated the accuracy of mean LIA measurements obtained with a depth camera by using rice cultivars and NILs that were expected to have differing leaf angles, as reported previously [2,13]. Tiller angle tended to be smaller in ‘Koshihikari’, KH-LIA3, and IR64-tac1, which all carry the *japonica* allele of *Tiller Angle Control 1* (*TAC1*), than in the others at all stages (Fig. 4A), in agreement with the findings of Okamura and Aoki [13]. A similar trend was observed in the mean LIA measured with the depth camera on 16 July and at full heading (Fig. 4B). KH-LIA3 has a quantitative trait locus (QTL), which results in a erect leaf [2]; its mean LIA tended to be lower than that of ‘Koshihikari’ on 22 August and 3 September.

**Fig. 5:**
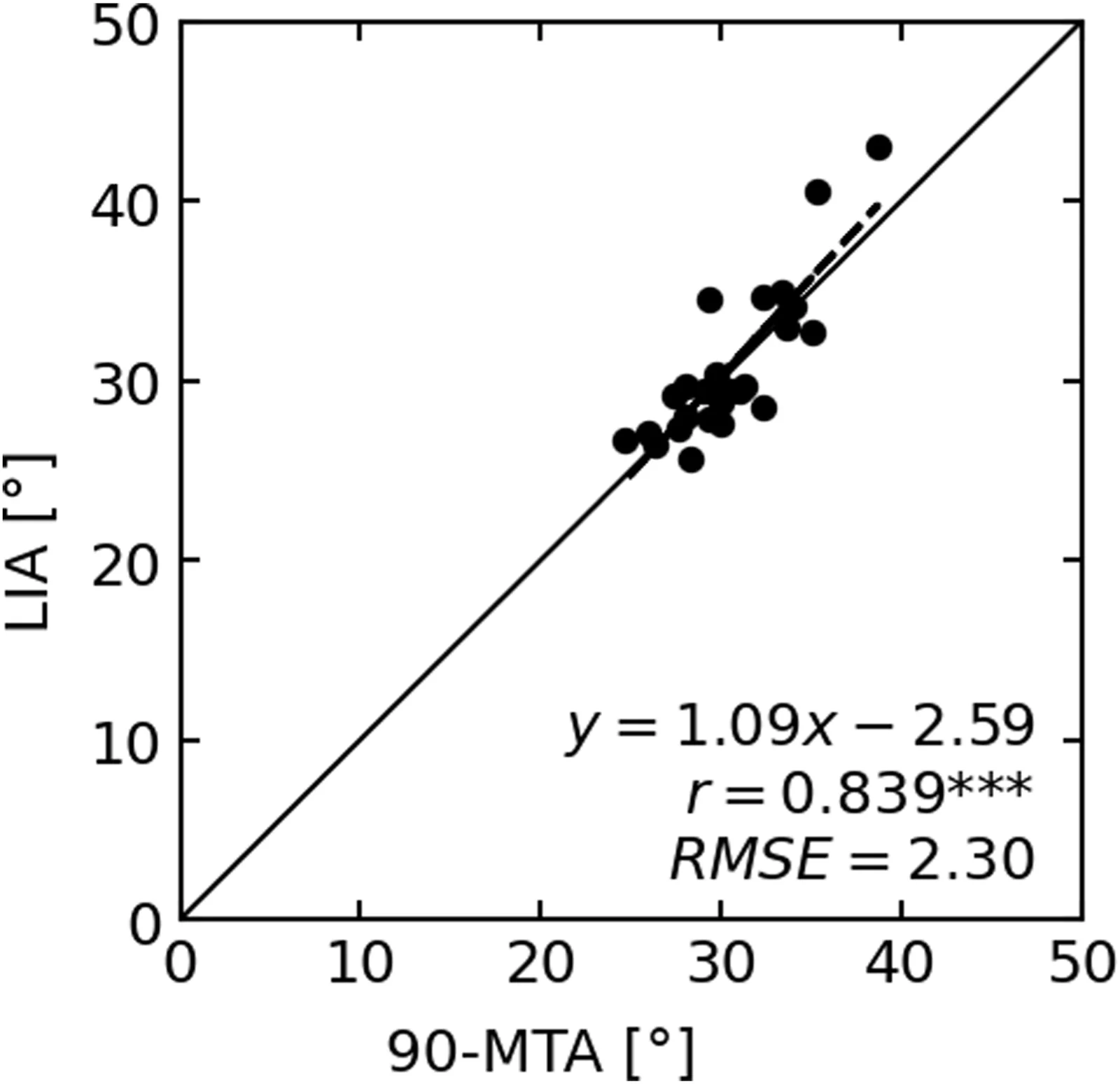
Comparison between leaf inclination angle (LIA) measured by a depth camera and mean tilt angle (MTA) measured by a plant canopy analyzer in rice. Diagonal solid line indicates 1:1. A linear regression equation is shown; the dashed line is the regression line. *r*, correlation coefficient. *RMSE*, Root Mean Square Error. ∗∗∗*P* < 0.001.

Although the PCA is a device for measuring leaf area, it also calculates the MTA of the measured targets. In rice canopies, where leaves account for a large proportion of the plant surface, MTA can be considered nearly equivalent to LIA. Therefore, we compared the MTA calculated by a PCA, with the mean LIA measured with the depth camera. Because LIA is measured relative to the vertical direction and MTA is measured relative to the horizontal direction, we compared LIA with the complementary angle of MTA (90° − MTA). Differences in 90 − MTA among cultivars and lines showed trends similar to those observed for mean LIA (Fig. 4C), and the two angles showed a strong correlation (Fig. 5).

**Fig. 6:**
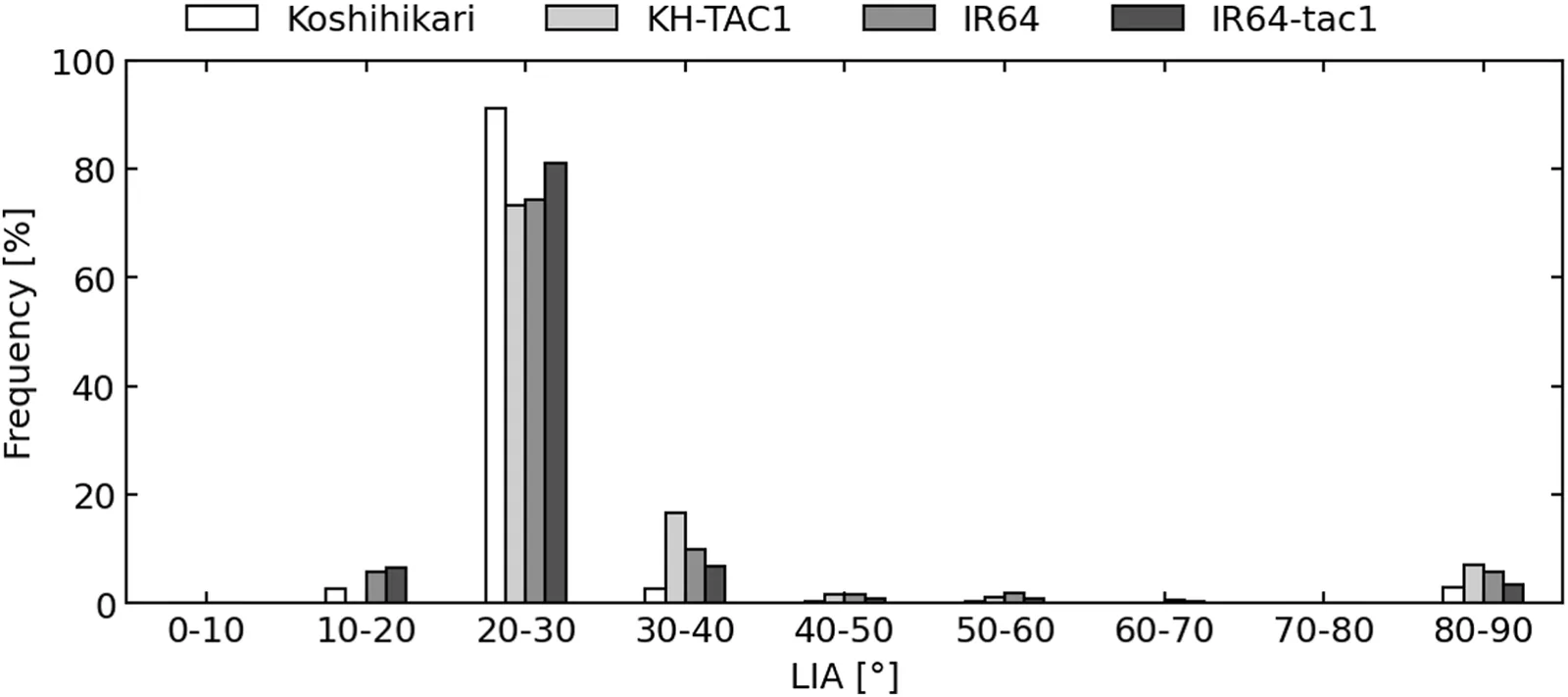
Distribution of leaf inclination angles (LIAs) in rice. Frequencies were calculated with all data of the three replicates.

**Fig. 7:**
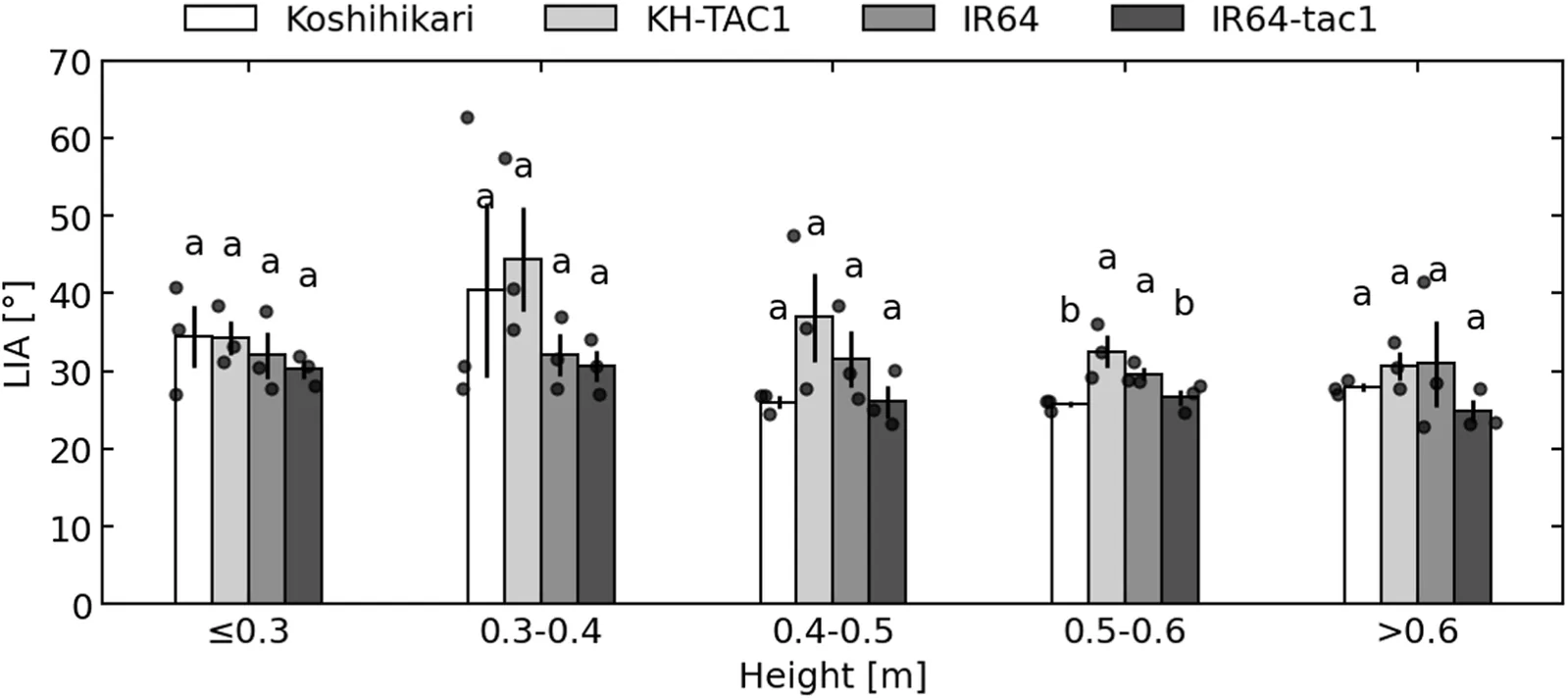
Mean leaf inclination angles (LIAs) at different canopy heights in rice. Values are means ± standard error (n = 3) with individual data points overlaid. Bars with a common letter within a cultivar/line are not significantly different (*P* ≥ 0.05, Tukey's test).

Measuring LIA with the depth camera allows us to obtain the frequency distribution of LIA and the mean LIA at different canopy heights. As the *TAC1* allele affects dry matter production as early as during the vegetative stage [13], we compared these values among the *TAC1* NILs and their parental cultivars on 16 July. The distribution of LIA was skewed toward angles greater than the peak, and extremely large angles (80°–90°) were also observed (Fig. 6). The proportion of leaves was tended to be lower in KH-TAC1 than in ‘Koshihikari’ in the 20°–30° class (the peak) but higher in KH-TAC1 in the 30°–40° class. In the comparison between ‘IR64’ and IR64-tac1, the opposite trend was observed (Fig. 6). In the comparison of different canopy heights, the difference in LIA between the NILs and their parental cultivars was largest at 0.4–0.6 m (Fig. 7).

### 3.3 Practical applications in other crops

**Fig. 8:**
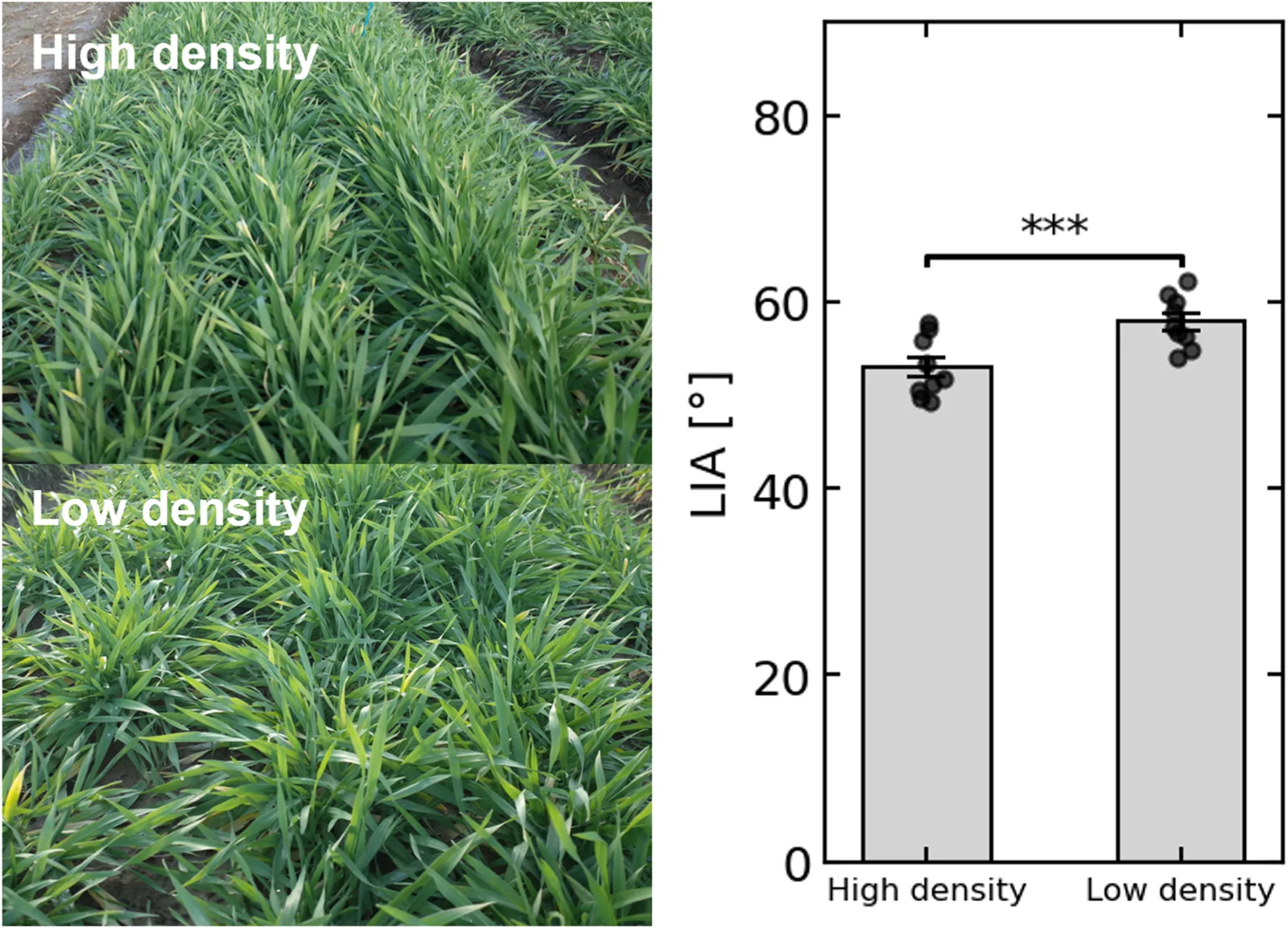
Comparison of leaf inclination angles (LIAs) at high and low density in barley. Values are means ± standard error (n = 9), with individual data points overlaid. In the two-way ANOVA in which the block is treated as a factor that does not take interaction effects into account, the cultivar effect was significant (∗∗∗*P* < 0.001).

Barley had more erect leaves at high seeding density than at low density 2 months after sowing (Fig. 8); this difference was statistically significant. Although this was a supplementary survey in which replicates were not randomized, LIA was also measured for soybean and wheat. LIA was measured in soybean at the R5 stage in 2024 and 2025. Cultivar differences in LIA were small but highly consistent in both years (Supplementary Fig. S2). A two-way ANOVA with year and cultivar as independent factors detected a significant cultivar effect. In wheat, ‘Yumechikara’ but not ‘Yukichikara’ had a prostrate growth habit, with leaves spreading close to the ground (Supplementary Fig. S3). This difference was clearly reflected in a significant difference in the LIA.

## 4 Discussion

### 4.1 Accuracy of our method

Our method allows us to measure the LIA with an inexpensive, compact depth camera and an ordinary laptop computer in a handheld manner. Validation demonstrated that, although simple, our algorithm is capable of capturing the inclinations of an object (white board) and single leaf surfaces (Fig. 3).

The whiteboard and single-leaf experiments demonstrated that our method can accurately measure absolute LIA values under artificially simplified measurement conditions. However, a complete ground truth for mean canopy LIA under field conditions cannot currently be obtained with existing technologies. Therefore, we compared the LIA of rice NILs and their parental cultivars, which were known to differ in plant architecture. As expected, cultivars and lines with larger tiller angles had larger LIA values (Fig. 4), in agreement with the findings of Okamura and Aoki [13]. KH-LIA3, carrying a QTL for LIA identified through representative leaf measurements [2], also had more erect leaves at the canopy level. Although this analysis requires caution because measurements were obtained only once per plot, the fact that the results are consistent with those of previous studies using the same NILs supports the validity of our approach in quantitatively capturing differences in rice canopy structure based on LIA. Additionally, the LIA measured by our method was strongly correlated with MTA calculated by using a PCA (Fig. 5), which essentially measures the same parameter as LIA. These results demonstrate the effectiveness of our method and an advancement of method by Uto et al. [12] into a more convenient handheld implementation while enabling the measurement of absolute angle values. However, it should be noted that, as with previous studies requiring more sophisticated equipment [[7], [8], [9], [10],^15^], these angle values have not been compared against a true ground truth.

We also quantified differences in LIA in barley, soybean and wheat (Fig. 8 and Supplementary Figs. S2 and S3). The measurements for soybean in 2025 and wheat were obtained from replicated plots that were not randomized among cultivars within the field; therefore, we cannot fully exclude the possibility that the observed differences reflected field heterogeneity rather than true cultivar differences. Nevertheless, our method successfully detects differences in plant architecture that are visually apparent yet have been difficult to quantify. We therefore concluded that the absence of randomization does not compromise the validity of our method, and that it is applicable not only to rice but also to a wide range of crop species.

When our method was repeatedly applied to the same canopy location under field conditions, the standard deviation was less than 1.5° (5%) (Supplementary Fig. S4). This deviation includes not only measurement uncertainties arising from factors such as lighting conditions and hand motion, but also changes in the target itself caused by wind. In any case, the repeatability of our method appears to be sufficient for detecting the differences in plant architecture described above.

### 4.2 Advantages of our method

A key advantage of our method lies not only in the fact that it does not require expensive equipment, but also in its ability to measure mean LIA within a very short time, both in terms of preparation and data acquisition. Our method allows measurements to be performed simply by starting a PC and connecting a stereo depth camera. The measurement itself requires only a single click, comparable to operation with a conventional RGB camera, allowing the entire process—from selecting the measurement location to acquiring the data—to be completed within several tens of seconds. In addition, because the computational procedure is highly straightforward, the results can be obtained and confirmed on site using a small, field-portable PC. Consequently, most of the time required for a series of measurements conducted on a given day is spent on traveling between measurement locations. In contrast, manual measurement using accelerometer-based sensors is itself very simple and rapid for each individual measurement. However, evaluating canopy-scale LIA requires the selection and measurement of a large number of representative leaves, which results in substantial labor and time requirements. In LiDAR-based 3D scanning, the scanning process itself may be completed within a few seconds when rapid systems are used. However, in practical applications to wheat and trees, scanners are typically fixed in position during data acquisition [[7], [8], [9], [10], [11]].Therefore, it is necessary to reinstall a mounting apparatus each time the measurement location is changed, which is expected to require substantial labor and time. Furthermore, it remains unclear whether such systems can be deployed within crop canopies, in particular under flooded conditions such as those in paddy rice fields. Methods based on pattern recognition using convolutional neural networks have also been developed to estimate the mean LIA and its distribution in trees from RGB images [15]. In the proposed approach, a fixed time-lapse camera is employed; however, if the method could be extended to handheld cameras, it might also be applicable to field-based crop research, similar to our approach. At present, however, there have been no reports demonstrating its applicability to crop species.

Our method enables not only the calculation of mean LIA but also the assessment of LIA distributions and mean LIA at different canopy heights (Fig. 6, Fig. 7). Our method successfully excludes non-leaf organs, such as panicles, by applying edge detection to RGB images (Fig. 2), whereas a PCA inherently includes the entire plant, including panicles, and reportedly can overestimate leaf area index in rice after heading [16]. In addition, as our method captures RGB images simultaneously with depth images, it is theoretically possible to exclude specific organs, including panicles, through post-processing of the RGB images.

Both our method and PCA share a limitation in that leaves masked by other leaves from the camera's viewpoint cannot be measured. In such cases, our top-down approach tends to exclude lower leaves, whereas PCA, which measures the canopy from below, tends to exclude upper leaves. Because upper leaves are generally more important for photosynthesis, this characteristic may represent an additional advantage of our method.

### 4.3 Flexibility of our method

Because the canopy structure of barley, soybean, and wheat differs from that of rice, we modified the vertical measurement range. At the early growth stage, barley and wheat had rosette leaves that lay close to the ground. Therefore, no layer was excluded below the base height. In theory, the concern with this approach is that ground surface angles may be included in LIA calculations. However, the canopy coverage ratio was high in our study, and statistically significant differences in LIA were detected (Fig. 8 and Supplementary Fig. S3). In contrast, in the measurement of canopies with rosette leaves and low canopy coverage ratio, additional approaches—such as removing ground surfaces through post-processing of the RGB images, rather than base height—would be required.

In comparison with the rice canopy, that of soybean has larger and more spreading leaves and closes in the upper layer [17]; in soybean, the LIA values might be more stable if measured at a specific height from the top of the canopy. Because leaf distributions and arrangements of the upper layer are important for photosynthetic efficiency in soybean [18], the LIA values only within the upper 0.2 m or 0.4 m of the canopy are appropriate for analyzing differences in productivity among soybean cultivars. Although the inter-cultivar differences in soybean were very small in this study, significant differences were detected by measuring 10 points per plot across three plots and repeating the measurements over 2 years (Supplementary Fig. S2). This was feasible owing to the short measurement time and low labor demand of our method.

In the measurements conducted on barley, soybean and wheat, the parameters for edge removal (i.e., the threshold and the number of pixels excluded near edges) were adopted from preliminary experiments conducted on rice. Although these settings appear to capture differences in canopy structure among these crop species, they influence which parts of the canopy are included in the calculation of mean LIA values: increasing sensitivity to edges enhances the contribution of larger leaves while reducing the influence of stems, panicles, and other non-leaf organs. Therefore, adjusting the parameters related to edge removal according to canopy architecture and research objectives may be a useful approach. These flexibility in adapting measurement settings to canopy structure is an advantage of our method.

### 4.4 Considerations of our method

We would also like to address the following two issues. (1) In the assessment of LIA distributions, a certain number of angles very close to horizontal far from the peak are counted. This is likely due to the generation of pixels with identical depth values during smoothing when depth images are acquired, where missing or outlier depth values are interpolated. Although this does not pose a considerable problem for comparisons of mean values, owing to the large number of pixels included in the calculation, frequency distributions should be considered with caution. (2) In rice, measurements were conducted under cloudy conditions, which are suitable for the PCA, but under sunny conditions the depth images were sometimes not captured properly, with unexpectedly large areas appearing at the same depth (Supplementary Fig. S5). The depth camera used in this study was equipped with an infrared pass filter to reduce depth noise caused by strong sunlight, but under extremely bright outdoor conditions the noise reduction might be limited. Therefore, we recommend that measurements be taken under cloudy conditions or with artificial shading. In soybean, barley, and wheat canopies, measurements were stable even under sunny conditions, suggesting that differences between upland crop fields and flooded rice fields may affect measurement stability. Both issues depend on the specifications of the depth camera used, and they might be resolved by using higher-specification ones. Although the compact depth camera used in this study was very convenient, this issue may be resolved through the selection of higher-performance cameras according to available budgets, as well as through future technological advancements.

## 5 Conclusion

We established a method to measure the mean LIA throughout the canopy by using only an inexpensive, compact stereo depth camera and a laptop computer. The accuracy of this method was validated through tests using a white board and single leaves. The mean LIA was strongly correlated with MTA, a related trait measured by PCA. Despite the lack of a complete ground-truth validation under filed conditions, our method was able to detect differences in LIA among cultivars, growth stages, and plant densities in rice, soybean, barley, and wheat. We hope that widespread adoption of this method, which can be readily applied alongside routine field experiments, will further advance research in crop science.

## Declaration of generative AI and AI-assisted technologies in the manuscript-preparation process

During the preparation of this work, the authors used M365 Copilot (Microsoft, Redmond, WA, USA) and ChatGPT (OpenAI, San Francisco, CA, USA) for programming support and English improvement. After using these tools, the authors reviewed and edited the content as needed, and they take full responsibility for the content of the published article.

## Author contributions

M. Okamura: Conceptualization, Methodology, Formal Analysis, Investigation, Writing-original draft, Visualisation, Conceptualization, Funding acquisition. R. Yamazaki: Methodology, Investigation, Review & Editing. N. Okamura: Methodology, Investigation, Review & Editing. Y. Shimazaki: Methodology, Investigation, Review & Editing. R. Sato: Investigation, Review & Editing. R. Morita: Investigation, Review & Editing. N. Aoki: Review & Editing, Supervision, Funding acquisition.

## Data availability

The datasets generated during and/or analyzed in this study are available from the corresponding authors on reasonable request. The program code (Program Registration Number at NARO: NARO-A53) is available upon request. It will be provided free of charge to public research institutions for research purposes, provided that the recipient enters into a licensing agreement with NARO and complies with the terms and conditions of use, including not authorizing any third party to use the program. For further details, please contact the corresponding author by email.

## Funding

This work was supported by 10.13039/501100001691JSPS KAKENHI Grant Numbers 23K26886 and 25K02001.

## Declaration of competing interest

The authors declare the following financial interests/personal relationships which may be considered as potential competing interests: Masaki Okamura reports financial support was provided by Japan Society for the Promotion of Science. Masaki Okamura has patent # INFORMATION PROCESSING DEVICE, INFORMATION PROCESSING METHOD, AND PROGRAM (JP, 7749275) issued to National Agriculture and Food Research Organization. If there are other authors, they declare that they have no known competing financial interests or personal relationships that could have appeared to influence the work reported in this paper.
