# Supplementary material for: A rapid and low-cost handheld method for measuring the mean leaf inclination angle of crop canopies
Source: Plant Phenomics. 2026 Jul 22;8(3):100260. doi: 10.1016/j.plaphe.2026.100260 (PMC13505371; doi:10.1016/j.plaphe.2026.100260)
Supplement: Supplementary file 1 [file mmc1.pdf]

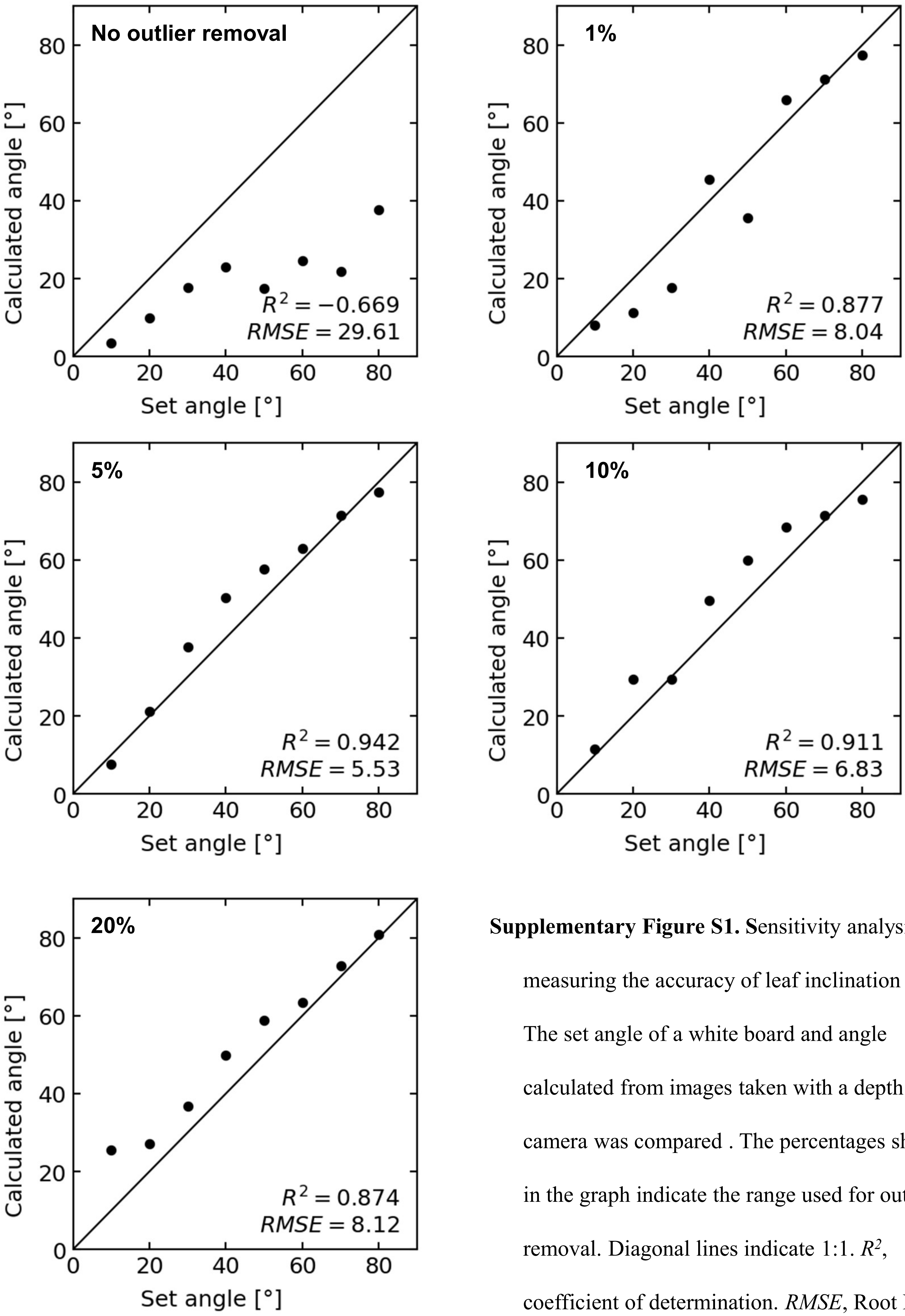

Supplementary Figure S1. Sensitivity analysis for measuring the accuracy of leaf inclination angles. The set angle of a white board and angle calculated from images taken with a depth camera was compared . The percentages shown in the graph indicate the range used for outlier removal. Diagonal lines indicate 1:1. R^2, coefficient of determination. *RMSE*, Root Mean Square Error.

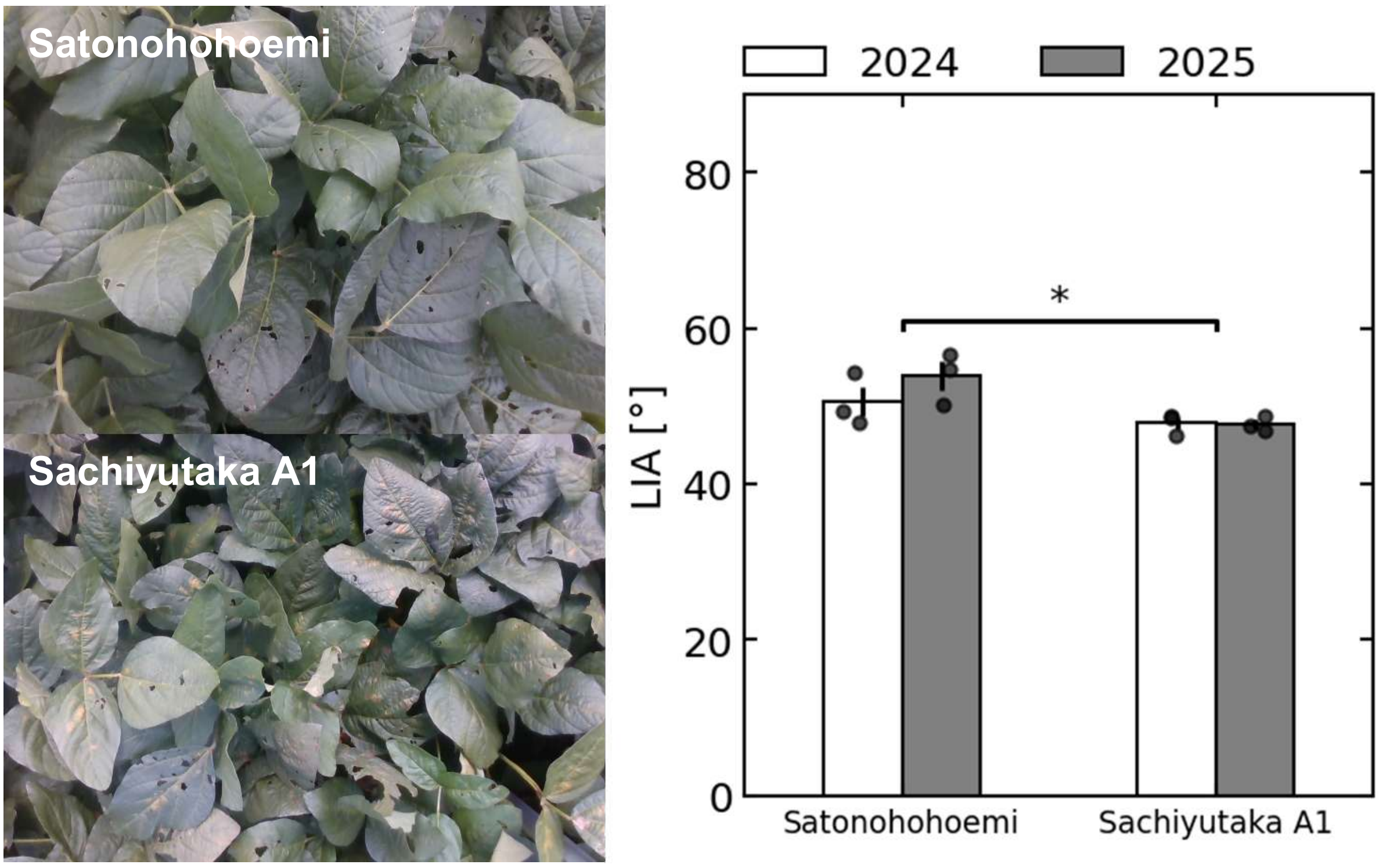

Supplementary Figure S2. Comparison of leaf inclination angles (LIA) between cultivars in soybean Soybeans (*Glycine max* (L.) Merr.). Pictures of Satonohohoemi (A) and Sachiyutaka A1(B), and LIA (C). Values are means ± standard error (n = 3) with means of each plot overlaid. In the two-way ANOVA, the cultivar effect was significant (*$P < 0.05$), whereas the year effect and the cultivar × year interaction were not significant. among cultivars and not significant among years ($P \geq 0.05$). Soybeans were sown in the Yawara experimental paddy field of NARO (36° 01′N, 140° 02′E, 10 m above sea level), Tsukubamirai, Ibaraki, Japan on 27 June 2024 and 18 June 2025, at a seeding density of 11 g m^{-2}. In 2024, the 22.4-m^2 plots were arranged in a randomized block design with three replicates. In 2025, the 17.5-m^2 plots were arranged with three replicates, without randomization among cultivars. Mean LIA was measured from 10 points per plot at the R5 stage (26 August 2024 and 28 August 2025 for 'Satonohohoemi', 11 September 2024 and 8 September 2025 for 'Sachiyutaka A1'). The measurement height was 0.5 m above the canopy top in 2024 and 0.3 m in 2025, and the base height was 0.2 m below the canopy top in 2024 and 0.4 m in 2025, allowing the LIA of the upper 0.2-m canopy layer in 2024 and 0.4-m canopy layer in 2025 to be measured.

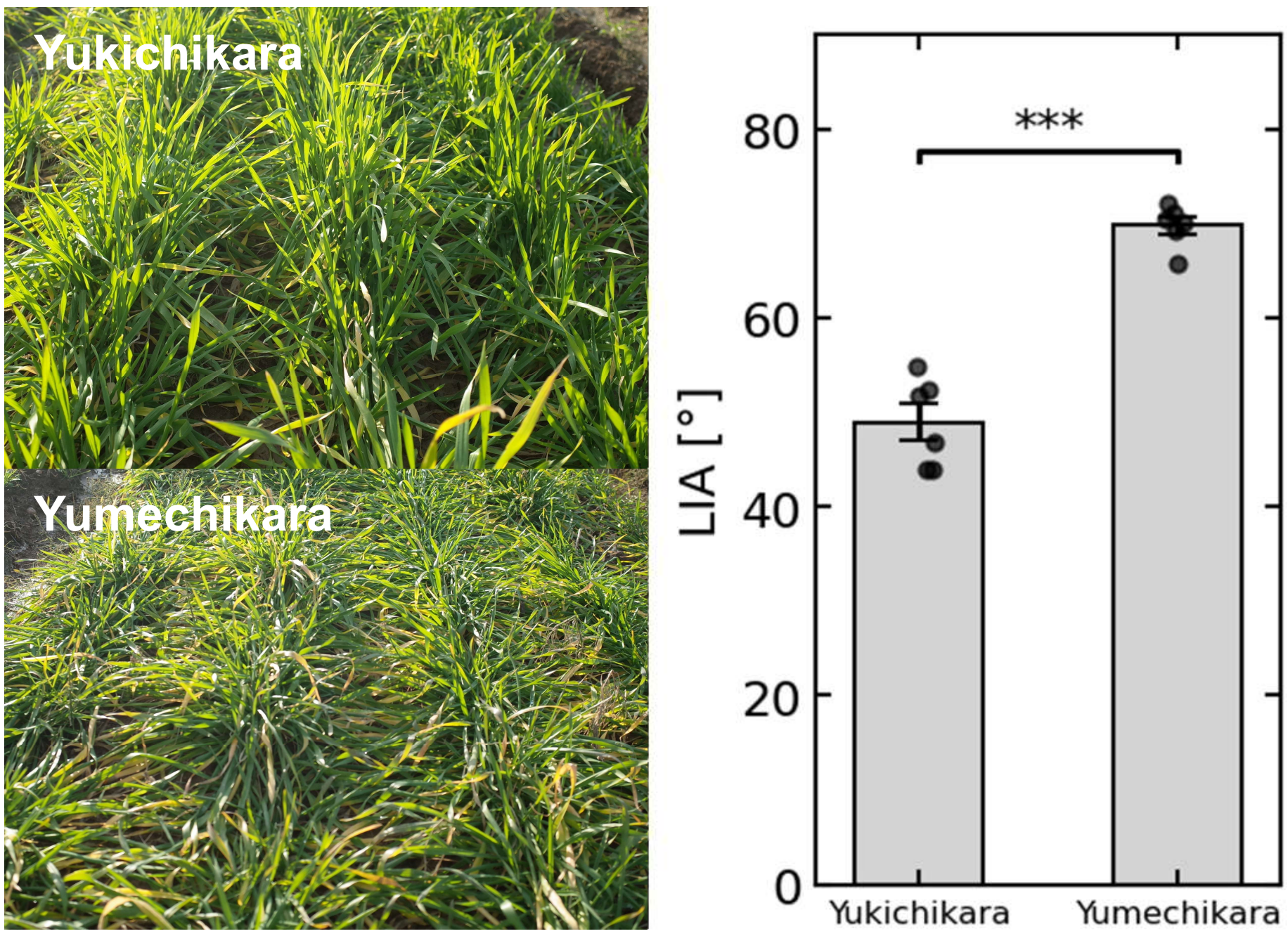

Supplementary Figure S3. Comparison of leaf inclination angles (LIA) between cultivars in wheat (*Triticum aestivum* L.). Pictures of Yukichikara (A) and Yumechikara (B), and LIA (C). Values are means ± standard error (n = 6) with individual data points overlaid. $^{***}P <$ 0.001(t-test). Wheat were sown in a drained paddy field at the Hokuriku Research Station of NARO on 3 October 2025. Inorganic fertilizers were applied as a basal dressing of 6 g N m^{-2}, 6 g P m^{-2}, and 6 g K m^{-2}. A single 9.6 m^2 plot had a seeding density of 8.8 g m^{-2}. On 11 December, mean LIA was measured, with six measurement points per plot. The measurement height was 0.8 m and the base height was not set because the plants had rosette leaves, and leaves touching the ground needed to be included at this growth stage.

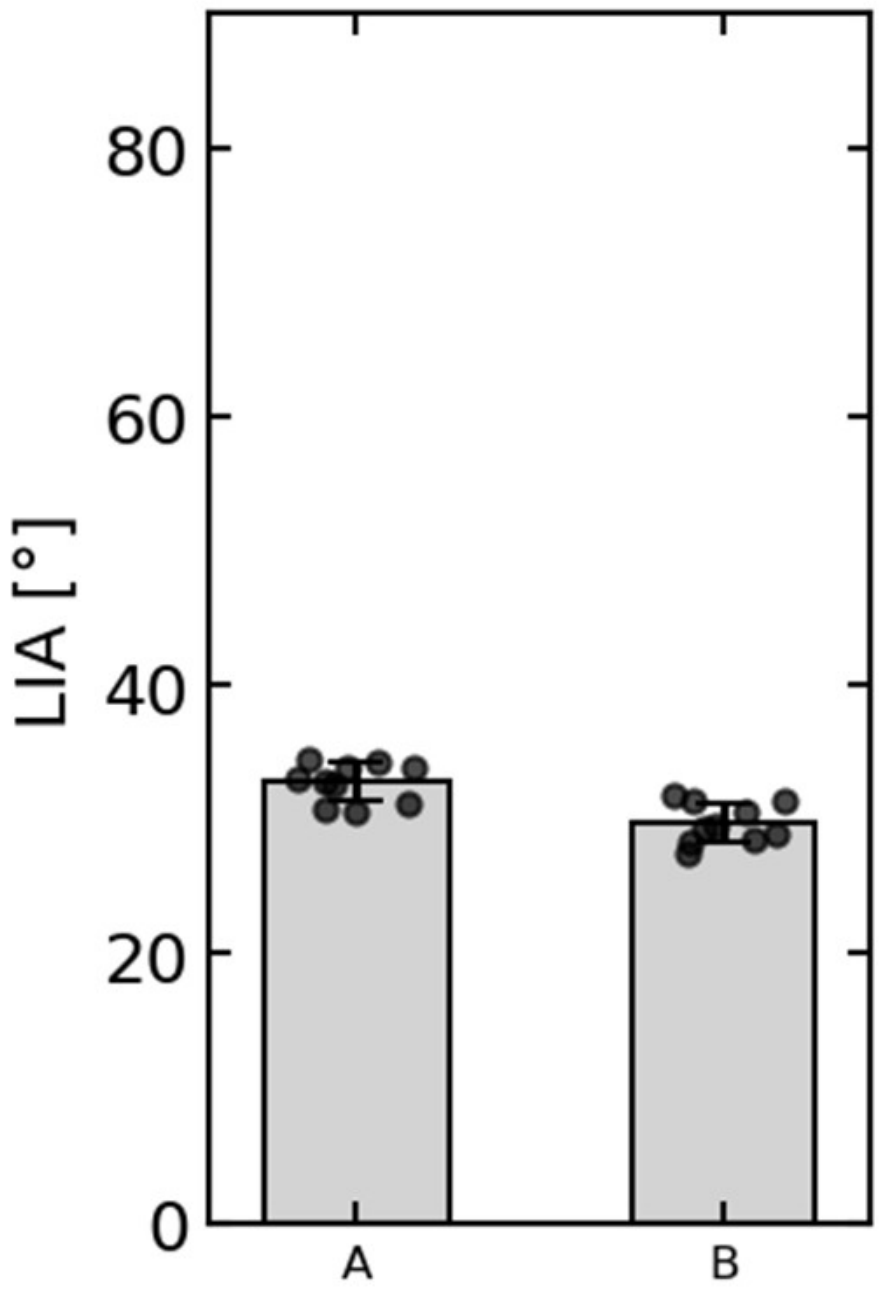

Supplementary Figure S4. Repeatability validation of the same canopy location in rice. LIA was measured 10 times at each of two locations (A and B) within the canopy on 6 July in 2026.Values are means ± standard deviation (n = 10) with individual data points overlaid. Rice cultivar 'Koshihikari' were trans planted in an experimental paddy field at the Hokuriku Research Station of NARO on 8 May 2026 . Fertilization conditions were the same as those used in 2024. The measurement height was 1.2 m and the base height was 0.2 m

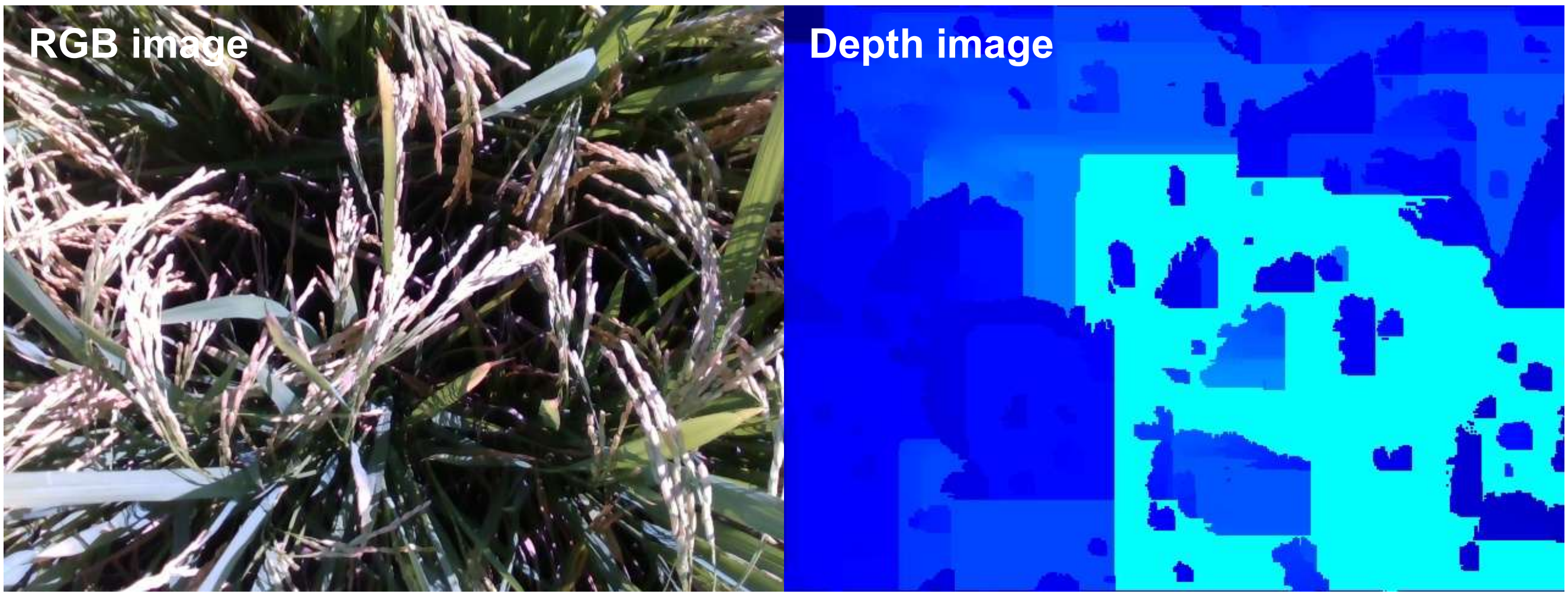

Supplementary Figure S5. An example of RGB and depth images acquired in a paddy field under strong direct sunlight. In the depth image, darker colors indicate regions closer to the camera.
